# Anti-Oxidative Effects of Rooibos Tea (Aspalathus linearis) on Immobilization-Induced Oxidative Stress in Rat Brain

**DOI:** 10.1371/journal.pone.0087061

**Published:** 2014-01-21

**Authors:** In-Sun Hong, Hwa-Yong Lee, Hyun-Pyo Kim

**Affiliations:** 1 Adult Stem Cell Research Center, Seoul National University, Seoul, Republic of Korea; 2 Department of Veterinary Public Health, Laboratory of Stem Cell and Tumor Biology, Seoul National University, Seoul, Republic of Korea; 3 Department of Biomedical Science, Jungwon University, Chungbuk, Korea; University of Kentucky, United States of America

## Abstract

Exposure to chronic psychological stress may be related to increased reactive oxygen species (ROS) or free radicals, and thus, long-term exposure to high levels of oxidative stress may cause the accumulation of oxidative damage and eventually lead to many neurodegenerative diseases. Compared with other organs, the brain appears especially susceptible to excessive oxidative stress due to its high demand for oxygen. In the case of excessive ROS production, endogenous defense mechanisms against ROS may not be sufficient to suppress ROS-associated oxidative damage. Dietary antioxidants have been shown to protect neurons against a variety of experimental neurodegenerative conditions. In particular, Rooibos tea might be a good source of antioxidants due to its larger proportion of polyphenolic compounds. An optimal animal model for stress should show the features of a stress response and should be able to mimic natural stress progression. However, most animal models of stress, such as cold-restraint, electric foot shock, and burn shock, usually involve physical abuse in addition to the psychological aspects of stress. Animals subjected to chronic restraint or immobilization are widely believed to be a convenient and reliable model to mimic psychological stress. Therefore, in the present study, we propose that immobilization-induced oxidative stress was significantly attenuated by treatment with Rooibos tea. This conclusion is demonstrated by Rooibos tea’s ability to (i) reverse the increase in stress-related metabolites (5-HIAA and FFA), (ii) prevent lipid peroxidation (LPO), (iii) restore stress-induced protein degradation (PD), (iv) regulate glutathione metabolism (GSH and GSH/GSSG ratio), and (v) modulate changes in the activities of antioxidant enzymes (SOD and CAT).

## Introduction

During cellular redox, the human body constantly generates free radicals (superoxide and hydroxyl radicals) and other reactive oxygen species (ROS) (hydrogen peroxide, nitric oxide, peroxynitrile, and hypochlorous acid) as a result of aerobic metabolism [1], [2]. Recent studies have suggested that long-term exposure to physiological or psychological stress is associated with the production of oxidative species, which cause the accumulation of oxidative damage to biomolecules (lipids, proteins, and DNA) in the brain, eventually leading to many neurodegenerative diseases [3], [4]. Many neurodegenerative diseases, such as Alzheimer's disease (AD) and Parkinson's disease (PD), are associated with an excessive production of ROS and free radicals [5], [6].

The brain is one of the most sensitive target tissues to oxidative stress because of its increased level of ROS and decreased level of antioxidants [7]. Moreover, ROS are constantly generated as by-products of normal metabolism and neurotransmitter metabolic processes. [8]. Consequently, ROS attack terminally differentiated neuronal cells, which are in a post-mitotic state unlike other cell populations (e.g., skin, blood, and connective tissue); therefore, neuronal cells are particularly sensitive to oxidative stress, leading to nerve damage [9]. In the case of excessive ROS production, endogenous defense mechanisms against ROS may not be sufficient to suppress ROS-associated oxidative damage [10].

Dietary antioxidants have been shown to protect neurons against a variety of experimental neurodegenerative conditions [11], [12]. Several natural beverages, in particular herbal teas, have potential against a variety of oxidative stress-induced neurodegenerative diseases. Rooibos tea, also known as Aspalathus linearis, is made from the flat acuminate leaves and yellow flowers; leaves are aromatic when dried and have traditionally been used as a medicine in South Africa [13]. Rooibos tea is caffeine-free (a benefit for pregnant women, children and caffeine-sensitive people) and contains very low levels of tannins [14]. Rooibos tea is an important source of dietary antioxidants, including flavonoids, dihydrochalcone glucoside, and aspalathin [15]. Numerous studies been conducted on the in vitro antioxidant activity of Rooibos tea with various types of extracts [16]–[19]. Rooibos tea has been shown to possess potent antimutagenic [18], [20], cancer-modulating [13], [17] and antioxidant activities by the regenerating coenzyme Q10 [21]. Therefore, an interesting question is whether rooibos tea, at a concentration commonly consumed in a beverage for humans, might have a protective effect on the oxidative stress caused by psychological stress and adaptation.

Various animal models for stress and stress-related phenomena have been developed and are frequently used to evaluate the protective effect of both natural products and synthetic compounds against stress. An optimal animal model for stress should have the features of a stress response and should be able to mimic natural stress progression. Various animal models for many different types of stressors, such as cold-restraint, electric foot shock, and burn shock, cause the oxidation of all molecular targets, including protein, DNA, and lipids, in tissues and organs [22]–[24]. However, most of these animal models of stress involve physical abuse in addition to the emotional or psychological aspects of stress. Animals subjected to chronic restraint or immobilization are widely believed to be a convenient and reliable model to mimic psychological stress. Numerous studies have investigated the effect of various antioxidant compounds on the chronic restraint or immobilization-induced stress model [25]–[27]. Therefore, in the present study, we investigated the effect of rooibos tea on oxidative stress-related alterations, such as stress-related metabolites, lipid peroxidative activity, glutathione metabolism, and enzymatic antioxidant systems, in chronic immobilization stress-exposed rats.

## Materials and Methods

### Rooibos Herbal Tea Preparations

Commercial Rooibos were purchased from Rooibos Ltd (Clanwilliam, South Africa). An aqueous extracts of rooibos were prepared by the addition of freshly boiled tap water to the commercial Rooibos (2 g/100 mL). These concentrations are customarily used for tea making purposes [28]. The mixture was allowed to stand for 30 min at room temperature, filtered and dispensed into water bottles.

### Treatment of Animals

Sprague Dawley (SD) rats (140–160 g body weight; Jungang animal Co., Korea) were housed in a temperature-controlled environment under a 12∶12 h dark:light cycle with food and water available ad libitum. The rats were divided into three groups (no stress, stress, and stress+ Rooibos) consisting of 10 rats per group. Each experimental group received the aqueous tea extracts for 4 weeks as their source of drinking water, while the control group received regular tap water. Fresh tea was prepared every day. Water is supplied free choice and they usually drink 15–20 ml a day (roughly 10 ml/100 g body weight/day). Animal experiments were approved by the ethics committee for animal experiments of Jungwon University (Permit Number: 2013-0410), and all efforts were made to minimize the number of animals used and their suffering.

### Immobilization Stress and Rooibos Tea Treatment

Rats (n = 10, each group) were immobilized for 1 hour per day for 4 weeks in tightly fitting ventilated plastic containers. The animals that were set free in their home cage in the absence of any stressors were used as controls. At the end of stress period, all animals were immediately decapitated, and their brains were rapidly removed and frozen at −80°C for following analysis. Blood was collected prior to the excision of brain, then treated with liquid nitrogen and stored at −80°C for further biochemical analysis. Rats were divided randomly into the following 3 groups: No stress (control), stress only, and stress+rooibos tea. Rooibos tea (3 g) was extracted with 200 ml of boiling water for 30 min and administered (20 ml/100 g body weight) 30 min prior to administering immobilization stress.

### 5-HIAA Measurement

HPLC was used to assay 5-HIAA. The HPLC was performed based on the method previously described [29] with some modifications. For monoamine analysis, an Agilent HC-C18 analytical column (250 mm×4.6 mm, 5 µm; Agilent, USA) was used. The mobile phase was composed of 20% methanol and 80% aqueous solution, which included 30 mM citric acid, 40 mM sodium acetate, 0.2 mM ethylenediaminetetraacetic acid (EDTA) disodium salt and 0.5 mM octanesulfonic acid sodium salt, at a flow rate of 1.0 ml/min and at pH value of 3.8. The level of 5-HIAA were detected using a Waters 474 scanning fluorescence detector (Waters, USA) with the excitation and emission wavelengths set at 280 nm and 330 nm, respectively.

### Free Fatty Acids (FFA) Measurement

Colorimetric assay was used for measuring plasma free fatty acids by means of Free Fatty Acid Quantification kit (Abcam, USA). In this assay, FFA are converted to their CoA derivatives, which are subsequently oxidized with the concomitant generation of color. C-8 (octanoate) and longer fatty acids can then be quantified by either colorimetric (spectrophotometry at 570 nm).

### Lipid Peroxidation Estimation

The level of malonyldialdehyde, as a substance that reacts with thiobarbituric acid, was determined in homogenates of the brain tissues and in plasma according to the method of Buege et al. [30] The 10% homogenates of tissues in 0.15 M KCl were centrifuged at 10,000 g for 30 min. To 0.5 ml of supernatant or 0.5 ml of serum 0.5 ml of 50% trichloroacetic acid were added and centrifuged again at 5,000 g for 5 min. After the final centrifugation, the tubes with 0.5 ml of supernatant and 0.5 ml of thiobarbituric acid covered with aluminium foil were incubated in a water bath at 90°c for 1 hour. The absorbance was read at 540 nm at room temperature against the blank and then concentration of thiobarbituric acid reactive substances (TBARS) was read from standard calibration curve, which was plotted using 1, 1, 3, 3′ tetra-ethoxy propane.

### Glutathione Assay in Tissue

Frozen tissue samples were homogenized (1∶10 w/v) in ice-cold sulfosalicylic acid (5%) previously bubbled with nitrogen gas for 10 min. Tissue extracts were bubbled with nitrogen gas for 10 s and centrifuged at 19,000 g at 4°C for 5 min. Supernatants were kept and used immediately to measure total glutathione (GSH-Eq = GSH+2GSSG) levels. GSH-Eq were determined by following the rate of reduction of 5,5′-dithiobis-2-nitrobenzoic acid (DTNB). Briefly, in a cuvette, potassium phosphate buffer (KPi 125 mM, pH 7.2; EDTA 6 mM), NADPH (0.3 mM), GR (50 U/mL), DTNB (6 mM), deionized water and 50 µL of sample were mixed. Change in absorbance per minute at 412 nm (ΔA412) was measured and GSH-Eq concentration in each sample was calculated from a standard curve (0–4 mM) [31]. Griffith's method with modifications [32] was used to quantify GSSG concentration. Sample extracts were mixed with 2VP (0.5 M), KPi buffer (0.5 M, pH 7.0) and adjusted to pH 7.0 with NaOH (1 M). Samples were incubated in the dark for 1 h. Then, deionized water, 150 µL of sample, NADPH (0.3 mM), GR (50 U/mL) and DTNB (6 mM) were mixed in a cuvette and absorbance was measured at 412 nm during 130 s. GSSG content was calculated comparing ΔA412 in the samples to a standard GSSG curve (0–1 µM). The GSSG/2GSH ratio was calculated using GSSG and GSH-Eq measurements and was expressed as GSSG:GSH-Eq.

### Glutathione Assay in Blood

To quantify whole blood glutathione after drawing a blood sample, 500 µL of whole blood was immediately transferred into a tube containing metaphosphoric acid. The solution was mixed and centrifuged (4000 rpm, 4°C, 10 minutes), the supernatant was collected. For the measurement of glutathione in erythrocytes, the whole blood was centrifuged for 10 min at 2,000 g and the plasma was aspirated. Then the equal volume of the 10% solution of metaphosphoric acid and the precipitate was mixed and kept at room temperature for 10 min. The sample was centrifuged at 4,000 g for 10 min and the supernatant was collected. Samples for reduced and oxidized glutathione were stored at –80°C until the analysis. Total glutathione and oxidized glutathione were measured by the enzymatic method of [33], which was modified and described by Kullisaar et [34]. The content of GSH was calculated as the difference between total glutathione and oxidized glutathione.

### Glutathione Peroxidase Assay (GSH-Px)

Glutathione peroxidase activity was measured by the method of Mohandas et al. [35]. The reaction mixture consisted of 1.5 ml phosphate buffer (0.1 M, pH 7.4), 0.1 ml sodium azide (1 mM), 0.05 ml glutathione reductase (1 IU/ml), 0.05 ml GSH (1 mM) 0.1 ml EDTA (1 mM), 0.1 ml NADPH (0.2 mM), 0.01 ml H2O2 (0.25 mM) and 0.1 ml 10% homogenate. The disappearance of NADPH at 340 nm was recorded at 25°C. Enzyme activity was calculated as nM NADPH oxidized/min/mg protein using molar extinction coefficient of 6.22×103/M cm.

### Glutathione Reductase Assay

Glutathione reductase activity was assayed by method of Fordyce et al. [36]. The reaction solution composed of 1.65 ml phosphate buffer: (0.1 M, pH 7.6), 0.1 ml EDTA (0.5 mM), 0.1 ml NADPH (0.1 mM) 0.05 ml oxidized glutathione (1 mM), and 0.1 ml 10% homogenate in a total volume of 2 ml. Enzyme activity was quantitated at 25°C by measuring disappearance of NADPH at 340 nm and was calculated as nM NADPH oxidized/min/mg protein using molar extinction coefficient of 6.22×103/M cm.

### Superoxide Dismutase (SOD) Assay

Total SOD (superoxide dismutase) activity was determined at room temperature according to the method of Misra et al. [37]. 10–30 µl of tissue homogenate was added to 3 ml of EDTA - sodium carbonate buffer (0.5 M) at pH 10.2. The reaction was started by adding 100 µl of epinephrine (30 mM in 0.1 M HCl) and the activity was measured at 480 nm for 4 min. One unit of SOD was defined as the amount of enzyme that inhibits by 50% the speed of oxidation of epinephrine.

### Catalase (CAT) Activity

Catalase activity was measured by the H_2_O_2_ degradation assay. Briefly, 50 µl tissue homogenate was added to a quartz cuvette containing 2.975 ml 0.05 M sodium phosphate buffer (pH 7.0) and 0.4 mM EDTA and 25 µl of 3% H_2_O_2_ was added to start the reaction. Catalase activity was determined by measuring the decrease in absorbance (H_2_O_2_ degradation) at 240 nm for 3 min and expressed as U/mg protein. One unit of catalase activity was defined as 1 µmol of H_2_O_2_ consumed/min.

### Statistical Analysis

The results are presented as a mean ± standard deviation. All the data were tested for their normal distribution. ANOVA for repeated measures was used to determine the significance of the differences in parameters. When significant ANOVA was found, the paired t-test for dependent data was used. Calculations were performed with the SPSS, Version 11.0 (SSPS Inc, Chicago, IL) statistical package. Statistical significance was defined as p<0.05.

## Results

### Rooibos Tea Administration Resulted in a Significant Reversal in the Stress-induced Inhibition in Body Weight Gain

**Figure 1:**
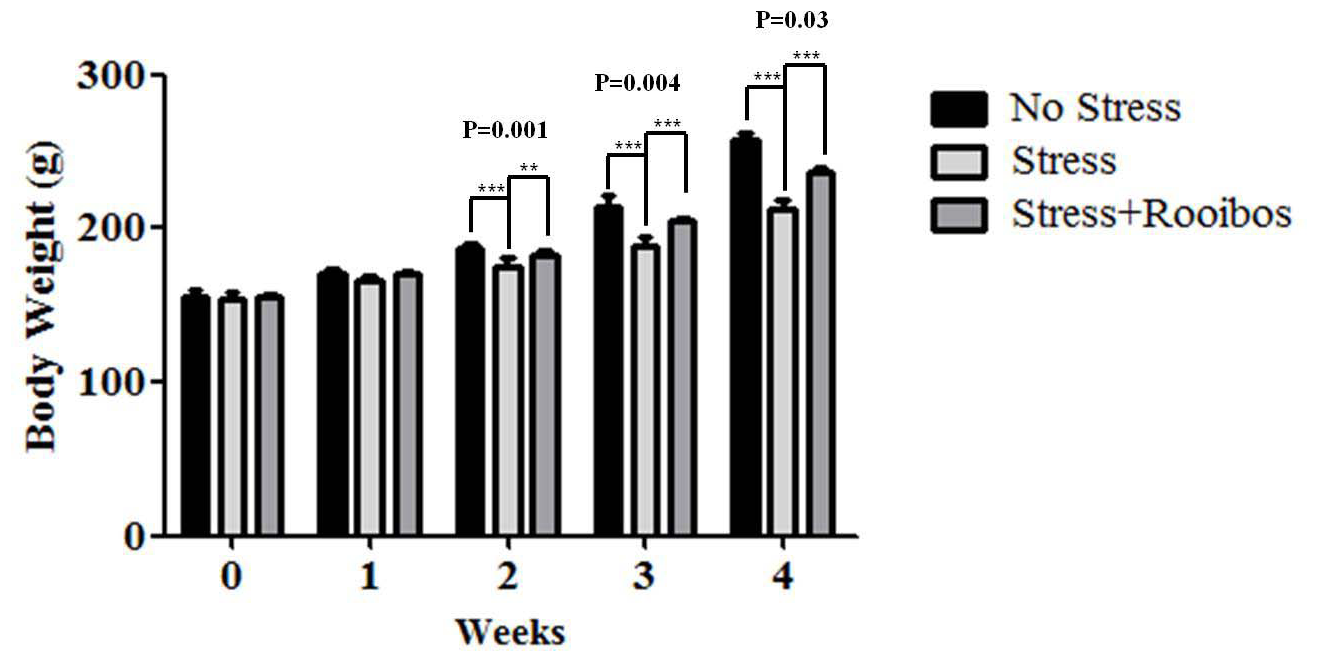
Continuous administration of Rooibos tea affects stress-induced changes in body weight. Rats were exposed to immobilization stress or immobilization stress plus Rooibos tea for 4 weeks. Rooibos tea was prepared according to a standard recipe (2 g Rooibos per 200 mL of freshly boiled water with a brewing time of 30 min). The body weight of animals was measured every 7 days. Rats in the non-stressed control group that received regular water had a normal weight accumulation, whereas the weight gain in the group that received the immobilization stress was significantly lower than the control group. The administration of Rooibos tea significantly slowed the immobilization stress-induced body weight loss. The results are expressed as the mean ± SD for 10 animals in each group. * P<0.05, ** P<0.01, and *** P<0.001.

A change in body weight is one of the well-known physical parameters that accompany the stress response. Therefore, to investigate the anti-oxidative effect of Rooibos tea on immobilization stress-induced body weight loss in a rat model, we monitored the body weight of rats from 4 weeks of age. As shown in Fig. 1, rats in the non-stressed control group that received regular water had a normal weight accumulation, whereas the weight gain in the group that received the immobilization stress was significantly lower than the control group. However, the administration of Rooibos-supplemented water significantly slowed the immobilization stress-induced body weight loss. As a result, at 4 weeks, the rats that received the immobilization stress lost on average 20% of their maximal body weight, whereas the body weight of the rats that received Rooibos-supplemented water with immobilization stress was reduced by 4%. Thus, treatment with the antioxidants in Rooibos tea significantly reversed the stress-induced weight loss in our immobilization stress animal model.

### The Effect of Rooibos Tea on Stress-related Metabolites

**Figure 2:**
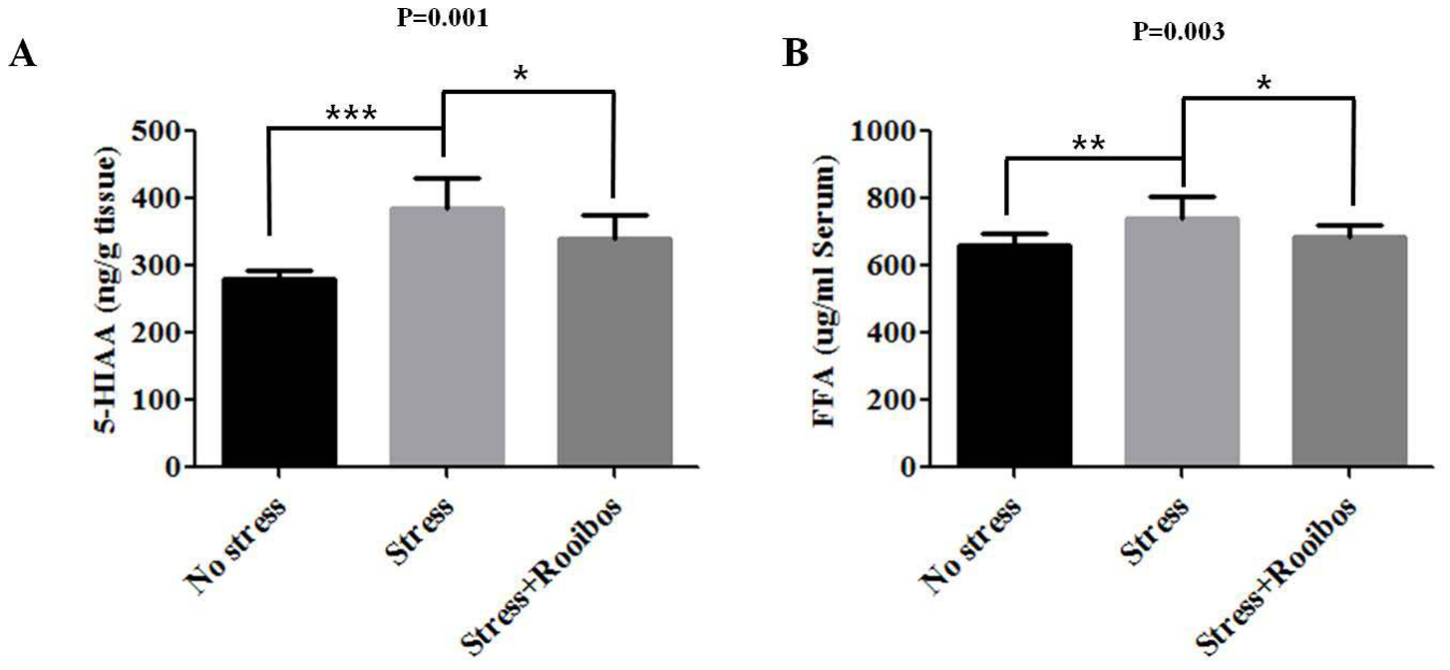
Effects of Rooibos tea on the stress-induced changes in 5-HIAA and FFA in stress-exposed rats. The experimental conditions were the same as described in Fig. 1. The levels of 5-HIAA (**A**) and FFA (**B**) were evaluated after rats were exposed to immobilization stress with or without Rooibos tea for 4 weeks. The exposure of rats to chronic stress increased the levels of 5-HIAA and FFA compared with the non-stressed control group, whereas prolonged treatment with Rooibos tea for 4 weeks attenuated the increase in the levels of 5-HIAA and FFA caused by chronic stress. The results are expressed as the mean ± SD for 10 animals in each group. * P<0.05, ** P<0.01, and *** P<0.001.

Reactions to stress are associated with the enhanced secretion of a number of hormones, including serotonin (5-hydroxytryptamine, 5-HT), which regulates the stress response pathway [38]. Serotonin can modulate the stress response by reducing the secretion of various stress hormones [39]–[41] and alleviating or eliminating aggressive behaviors [42]. In previous studies, the concentration of 5-hydroxyindoleacetic acid (5-HIAA), the primary metabolite of serotonin, in the brain was significantly increased by exposure to chronic stress [43], [44]. Therefore, to investigate the anti-stress effect of Rooibos tea on the stress-induced changes in 5-HIAA levels in a rat model, we measured the levels of 5-HIAA with and without Rooibos tea administration. Chronic stress caused increased 5-HIAA levels compared with the control group, whereas prolonged treatment with Rooibos tea for 4 weeks counteracted the increase in the 5-HIAA level caused by chronic stress in the brain extracts (Fig. 2A).

Non-esterified fats are known as free fatty acids (FFA) that circulate in the bloodstream and are predominantly bound to albumin [45]. Several groups have found that FFA may enhance oxidative stress by increasing ROS production in in vitro studies [46], [47]. In contrast, no in vivo studies have addressed this relationship. Therefore, to determine the potential link between stress and increased FFA production, rats were exposed to immobilization stress with and without Rooibos tea for 4 weeks. The exposure of rats to chronic stress increased FFA production compared with the control group, whereas prolonged treatment with Rooibos tea for 4 weeks attenuated the increase in FFA production caused by chronic stress (Fig. 2B).

### Lipid Peroxidative Activity in Stress-exposed Rats with and without Rooibos Tea Administration

**Figure 3:**
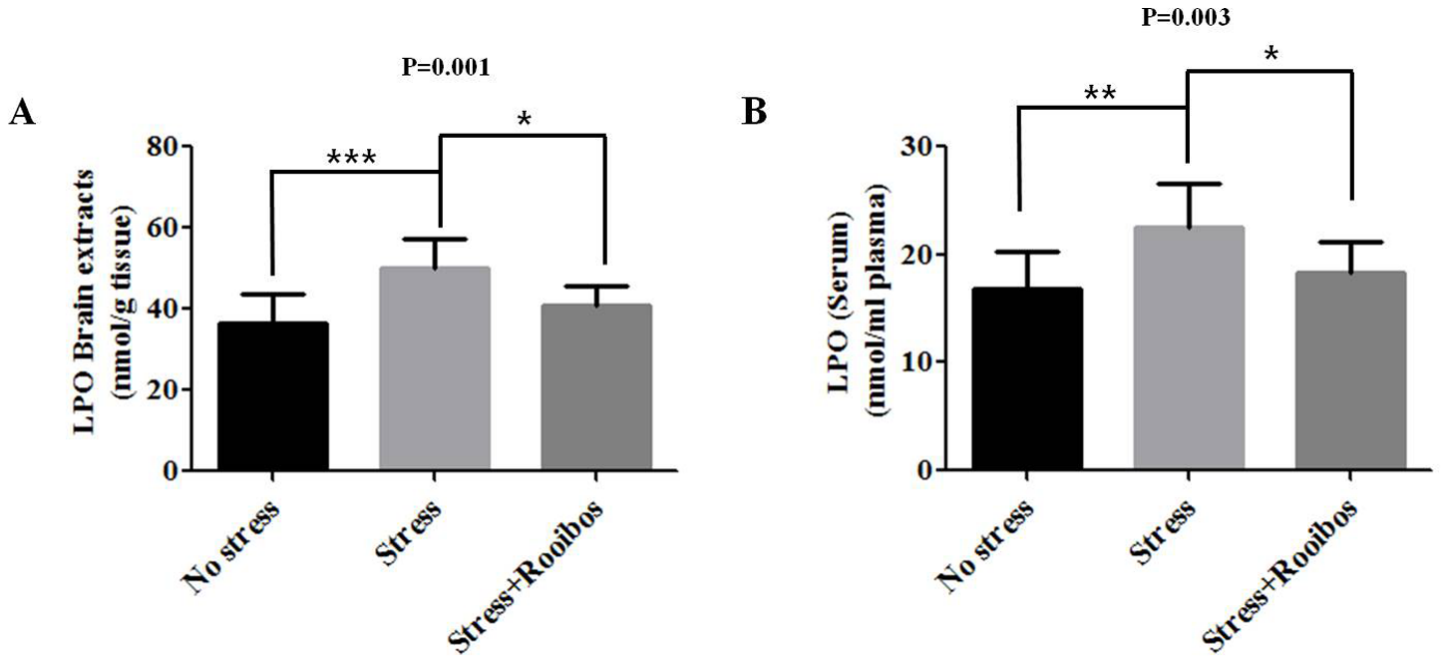
Effects of Rooibos tea on the stress-induced changes in LPO in stress-exposed rats. The experimental conditions were the same as described in Fig. 1. The levels of LPO in the brain (**A**) and bloodstream (**B**) were evaluated after rats were exposed to immobilization stress with or without Rooibos tea for 4 weeks. The administration of Rooibos tea to immobilization stress-exposed rats decreased the level of LPO in both brain extracts and serum. The results are expressed as the mean ± SD for 10 animals in each group. * P<0.05, ** P<0.01, and *** P<0.001.

Under oxidative stress conditions, fatty acids, especially unsaturated fatty acids, can undergo lipid peroxidation (LPO) to form complex reactive aldehyde species that are cytotoxic through their ability to react with proteins, DNA, and lipids [48], [49]. Lipid peroxidation (LPO), a well-established oxidative stress marker, can be used as an indicator of oxidative damage in cells and tissues [50]. In the present study, we evaluated the level of LPO in brain extracts and serum as an oxidative stress marker to evaluate changes in oxidative stress induced by immobilization stress with and without Rooibos tea administration. The exposure of rats to chronic stress increased the LPO concentration compared with the control group. However, the administration of Rooibos tea to immobilization stress-exposed rats caused a decrease in the level of LPO in both brain extracts (Fig. 3A) and serum (Fig. 3B).

### Rooibos Tea Prevents Immobilization Stress-induced Protein Degradation (PD)

**Figure 4:**
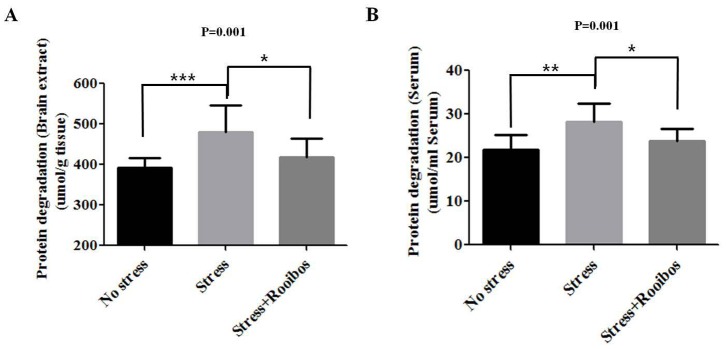
Effects of Rooibos tea on the stress-induced changes of PD in stress-exposed rats. The experimental conditions were the same as described in Fig. 1. The levels of PD in the brain (**A**) and red blood cells (**B**) were evaluated after rats were exposed to immobilization stress with or without Rooibos tea for 4 weeks. Prolonged treatment with Rooibos tea for 4 weeks attenuated the PD caused by chronic stress. The results are expressed as the mean ± SD for 10 animals in each group. * P<0.05, ** P<0.01, and *** P<0.001.

Proteins are one of the prime targets for oxidative damage. Protein oxidation is defined as the reaction of oxygen species (hydrogen peroxide, superoxide, and hydroxyl radical) and reactive nitrogen species (peroxynitrite and nitric oxide) with the backbone of the polypeptide and specific amino acid side chains [51]. Because proteins are responsible for most biological functions in cells, the degradation of oxidized proteins can lead to diverse functional consequences [52], [53]. Therefore, to investigate the anti-stress effect of Rooibos tea on stress-induced protein degradation in a rat model, we measured the levels of protein degradation in brain extracts and red blood cells. The exposure of rats to chronic stress increased protein degradation compared with the control group, whereas prolonged treatment with Rooibos tea for 4 weeks attenuated the protein degradation caused by chronic stress (Fig. 4A and B).

### The Effect of Rooibos Tea on Glutathione Metabolism and its Related Enzymes in Stress-exposed Rats

**Figure 5:**
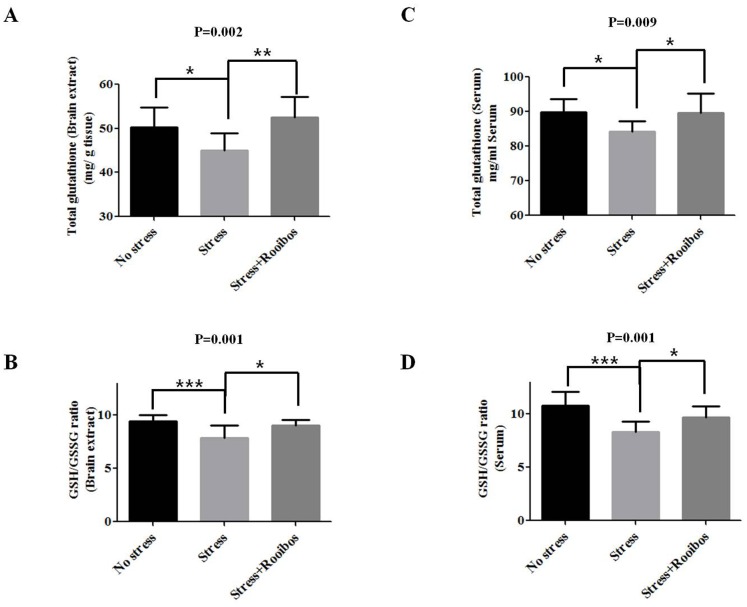
Effects of Rooibos tea on the stress-induced changes of glutathione metabolism in stress-exposed rats. The experimental conditions were the same as described in Fig. 1. The levels of GSH (**A, C**) and the GSH/GSSG ratio (**B, D**) in the brain and bloodstream were evaluated after rats were exposed to immobilization stress with or without Rooibos tea for 4 weeks. Prolonged treatment with Rooibos tea for 4 weeks counteracted the decrease in total GSH levels and the GSH/GSSG ratio caused by chronic stress in brain extracts and red blood cells. The results are expressed as the mean ± SD for 10 animals in each group. * P<0.05, ** P<0.01, and *** P<0.001.

The tripeptide glutathione (GSH) is the most abundant thiol compound in mammalian cells and functions as an antioxidant, preventing the damage caused by oxidative stress in the brain [54]–[56]. GSH is an important component for the cellular defense system against oxidative stress. A high level of intracellular GSH protects against a variety of oxidative stressors, such as free radicals, H_2_O_2_ or nitric oxide [54]. In a healthy cell, glutathione normally exists as the reduced form (GSH), but GSH is converted into its oxidized form (GSSG) when cells are exposed to increased levels of oxidative stress. Therefore, the ratio of GSSG to GSH has been used as a sensitive index of oxidative stress in biological systems [57]. To investigate the anti-oxidative effect of Rooibos tea on stress-induced changes in the GSH level in a rat model, we measured the levels of total GSH and the ratio of GSH/GSSG with and without Rooibos tea administration. Chronic stress caused a decrease in the total GSH level and GSH/GSSG ratio compared with the control group, whereas prolonged treatment with Rooibos tea for 4 weeks counteracted the decrease in the total GSH level and GSH/GSSG ratio caused by chronic stress in brain extracts and red blood cells (Fig. 5).

**Figure 6:**
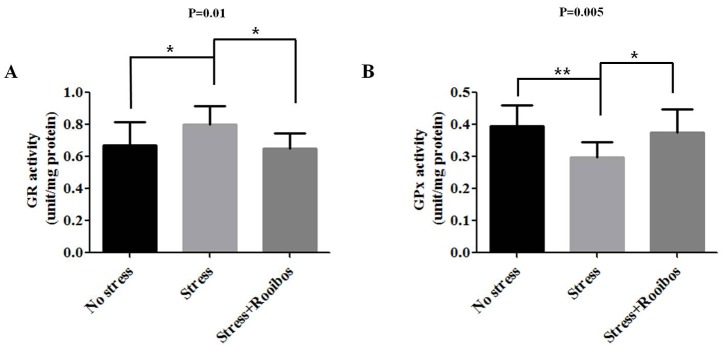
Effects of Rooibos tea on the stress-induced changes in GR and GPx activities in stress-exposed rats. The experimental conditions were the same as described in Fig. 1. The levels of GR (**A**) and GPx (**B**) in the brain were evaluated after rats were exposed to immobilization stress with or without Rooibos tea for 4 weeks. The chronic stress-induced stimulatory and inhibitory effects on these enzymes in brain extracts were attenuated by treatment with Rooibos tea for 4 weeks. The results are expressed as the mean ± SD for 10 animals in each group. * P<0.05, ** P<0.01, and *** P<0.001.

Glutathione reductase (GR) is the enzyme that is responsible for recycling oxidized GSSG to the antioxidant form (GSH) within most cells by using NADPH as an electron donor [58] and has been shown to be up-regulated following oxidative stress and cellular injury [56], [59]. For example, the enzymatic activity of GR was up-regulated under oxidative stress in human keratinocytes and fibroblasts [59]. Glutathione peroxidase (GPx), a potent anti-oxidative enzyme, catalyzes the reduction of peroxides by using glutathione as the electron donor. Oxidative stress has been shown to significantly reduce GPx activity in several pathological animal models [60]. Therefore, to investigate the anti-stress effect of Rooibos tea on stress-induced changes in GSH and GPx activities in a rat model, we measured the activities of GR and GPx with and without Rooibos tea administration. Chronic stress increased and decreased the activities of GR and GPx, respectively, compared with the control group. The chronic stress-induced stimulatory and inhibitory effects on these enzymes in brain extracts were attenuated by treatment with Rooibos tea for 4 weeks (Fig. 6).

### The Effect of Rooibos Tea on the Activities of Superoxide Dismutase (SOD) and Catalase (CAT) in Stress-exposed Rats

**Figure 7:**
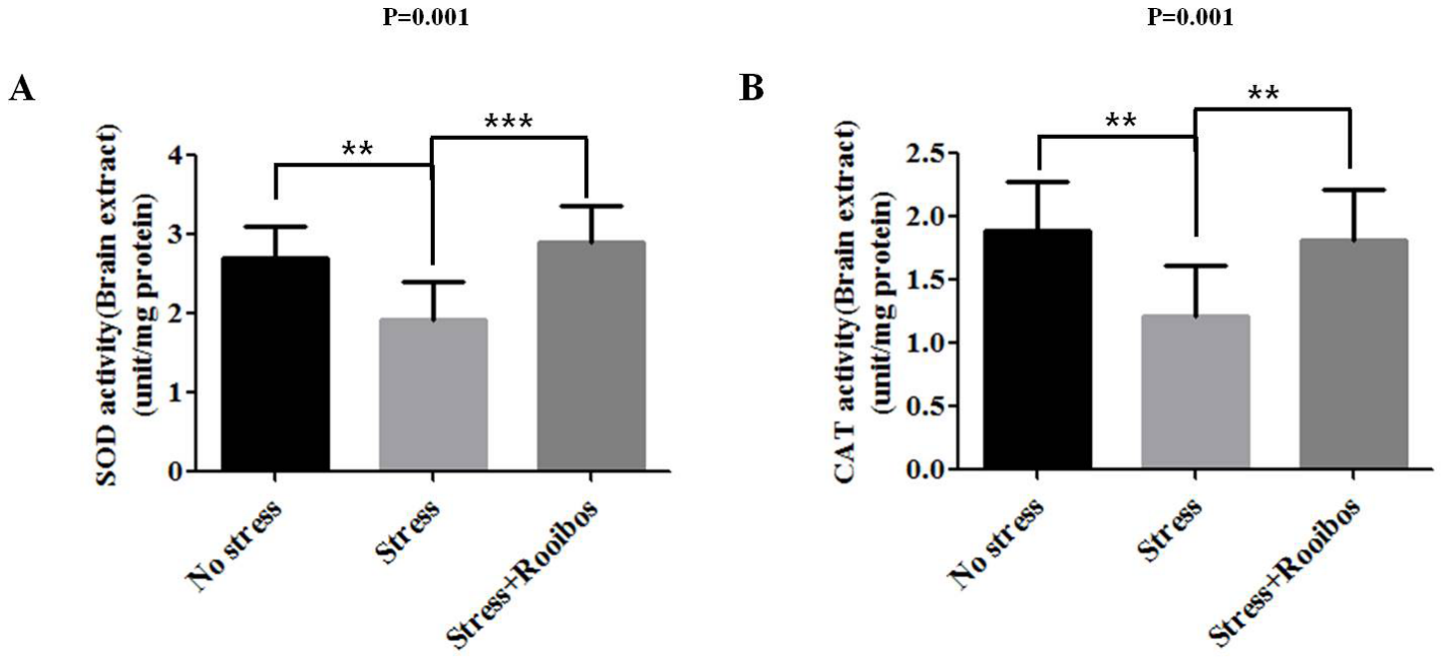
Effect of Rooibos tea on the activity of SOD and CAT in stress-exposed rats. The experimental conditions were the same as described in Fig. 1. The levels of SOD (**A**) and CAT (**B**) in the brain were evaluated after rats were exposed to immobilization stress with or without Rooibos tea for 4 weeks. The chronic stress-induced inhibitory effects on these enzymes in brain extracts were attenuated by treatment with Rooibos tea for 4 weeks. The results are expressed as the mean ± SD for 10 animals in each group. * P<0.05, ** P<0.01, and *** P<0.001.

Glutathione metabolism and its related enzymes are one part of the cellular defense system against oxidative stress. Other potent anti-oxidative enzymes, such as SOD and CAT, are also thought to be involved in the cellular protection against oxidative stress [61], [62]. SOD, which catalyzes the breakdown of superoxide (O^2−^) into hydrogen peroxide (H_2_O_2_) and molecular oxygen (O_2_) in the cellular defense system against oxidative stress, is one of the most potent antioxidant enzymes [63]. CAT is a ubiquitous anti-oxidative enzyme found in most aerobic cells exposed to oxygen. CAT is involved in the cellular protection against oxidative stress by catalyzing the decomposition of hydrogen peroxide (H_2_O_2_) to water (H_2_O) and oxygen (O_2_) [64]. Chronic stress decreased the activities of SOD and CAT compared with the control group. The chronic stress-induced inhibitory effects on these enzymes in brain extracts were attenuated by treatment with Rooibos tea for 4 weeks (Fig. 7).

## Discussion

An intense stress response results in the generation of free radicals and other reactive oxygen species (ROS), such as hydroxyl radical (HO), hydrogen peroxide (H_2_O_2_), and superoxide anion radical (O^2−^), that results in lipid peroxidation, especially in cell membranes and can alter membrane integrity, leading to tissue injury [65]. Previous studies have suggested that exposure to chronic psychological stress is related to increased free radicals (oxidative stress), and thus, long-term exposure to high levels of psychological stressors may cause many different types of neurodegenerative diseases, such as Alzheimer’s, Parkinson’s and Huntington’s disease [66]–[68].

Compared with other organs, the brain appears especially susceptible to excessive oxidative stress. The brain is one of the most metabolically active organs in the body. Although this organ comprises only 2% of the total body weight, the cells of the human brain demand at least 20% of the body's available oxygen supply [55]. This demand suggests the potential generation of a large amount of ROS in the process of oxidative phosphorylation during the production of ATP. Moreover, high levels of iron concentration have been reported in certain regions of the brain that are able to catalyze the generation of ROS [69]. The brain might be more susceptible to ROS because it contains high levels of unsaturated fatty acids, which are the main targets of free radicals and cellular lipid peroxidation. Moreover, the brain contains only low to moderate levels of antioxidant enzyme activities, such as superoxide dismutase (SOD), catalase (CAT), and glutathione peroxidase (GPx), compared with other organs [46]. Under normal circumstances, multiple endogenous protective mechanisms against oxidative stress have developed in mammalian cells to limit free radicals and the damage caused by them. However, in the case of excessive generation of ROS in the brain, endogenous defense mechanisms may not be sufficient to scavenge or detoxify ROS, and additional protective mechanisms through exogenous dietary antioxidants are of great importance [10]. Dietary antioxidants, such as glutathione, taurine, selenium, zinc, vitamin C, and polyphenols, help to limit the excessive generation of ROS.

Numerous fruits and vegetables with antioxidative and radical-scavenging properties have been studied for the purpose of preventing many oxidative stress-related diseases [70]. Several comparative advantages of using natural products to prevent oxidative stress are the following: i) low or no toxicity; ii) the unusual mixture of multiple antioxidants in the product; iii) ability to react to most or all types of ROS; and iv) easy accessibility. Herbal tea is one of the most popular and widely consumed non-alcoholic beverages among the different types of natural products [71], [72]. The protective effect of these herbal teas against oxidative stress may be predominantly due to the presence of polyphenols [72]. Recently, the role of polyphenols, particularly flavanoids, has been explored for their neuroprotective effects against pathological conditions [73]–[75]. In particular, Rooibos tea is considered a good source of antioxidants due to the larger proportion of polyphenolic compounds, including flavonoids and phenolic acids, that are potent antioxidants and free radical scavengers [76]. Rooibos has been shown to prevent chemically induced liver damage [77], inflammation [78], lipid oxidation [79], hyperglycemia [80], and oxidative stress [81]. These antioxidant effects of Rooibos tea may be related to the presence of antioxidant polyphenol compounds, including aspalathin (dihydrochalcone C-glucoside aspalathin), nothofagin (C–C linked dihydrochalcone glucoside), and other flavonoids (isoorientin and orientin, which are both oxidation products of aspalathin that are characterized by higher stability at high temperatures and under varying pH conditions) [82]. While we have confirmed that drinking Rooibos tea protects against oxidative effects induced by immobilization stress in rats, the exact mechanism of action of Rooibos is still largely unknown. Although some of Rooibos-mediated mechanism have been characterized, they have been confined to one or a few of these features in which a more comprehensive profile of the Rooibos-mediated mechanisms is still lacking. Previous studies have suggested that Rooibos increases stress resistance, probably mediated via a regulation of the DAF-16/FOXO insulin-like signaling pathway [83]. Hence, further studies are needed to assess the effects of Rooibos on the expression of stress related proteins such as p53, p38, heat shock proteins (HSP), SOD, and Gpx to corroborate our findings.

As chronic restraint or immobilization stress is believed to be the most potent psychological stress model without physical abuse in rodents and has a comparative effect in humans, this animal model for psychological stress was used in the present study [84]. Chronic restraint or immobilization stress was reported to be a good model for psychological stress-meditated alterations in the balance of oxidant–antioxidant in brain tissue [27]. In addition, chronic immobilization stress has been reported to show some symptoms of psychological stress, such as impaired motor activity and anxiety [85], pain perception [86], and depression-like behaviors [87] in animals. In the present study, chronic immobilization stress caused significant increases in indicators of oxidative stress, such as lipid peroxidative activity, protein degradation (PD), glutathione metabolites, and anti-oxidative enzymes, in the brain.

GSH plays an important role in detoxification in the brain [54]–[56]. In our study, brain GSH levels were decreased in all stress groups. Stress reduces GSH levels and leads to increased levels of ROS in rat brains [88]. Evidence has been presented that the enzymatic antioxidant defense system against hydrogen peroxide (H_2_O_2_), which is the most powerful toxic molecule to the brain, is primarily mediated by the GSH system [55]. In a healthy cell, glutathione normally exists as the reduced form (GSH), but GSH is converted into its oxidized form (GSSG) when cells are exposed to increased levels of oxidative stress. Therefore, the ratio of GSSG to GSH has been used as a sensitive index of oxidative stress in biological systems [57]. In this study, treatment with Rooibos tea restored the oxidative stress-induced reduction of GSH levels and the GSH: GSSG ratio. As fatty acids, especially unsaturated fatty acids, can undergo lipid peroxidation (LPO) under oxidative stress conditions, LPO is a well-established marker for oxidative stress or oxidative damage [50]. Rooibos tea has been previously reported to reduce age-related LPO accumulation (measured as TBARS) in the brains of rats consuming the herbal tea for 21 months [89]. Similarly, the present study showed that rooibos tea treatment was also found to be highly protective against LPO accumulation in the brains of rats subjected to immobilization stress. Antioxidant enzymes with radical scavenging and repair activities counteract ROS and ROS-induced damage triggered by oxidative stress. The combined anti-oxidative effects of SOD and CAT are supposedly sufficient to eliminate oxygen and hydrogen peroxide and protect cellular components against the more reactive hydroxyl radical [90]. Previously, Suresh et al. [91] reported that a flavonoid-rich tropical herbal plant reversed and caused a significant increase in the activities of SOD and CAT. Therefore, in the present study, we propose that immobilization stress-induced inhibitory effects on the activities of these anti-oxidative enzymes were attenuated by treatment with Rooibos tea, and the activities of these enzymes could be the protective leverage for the psychological stress-induced free radicals or reactive oxygen species.

In conclusion, the present study suggests that antioxidant-rich rooibos tea showed efficient protective action against immobilization-induced oxidative stress in rats. This conclusion is demonstrated by rooibos tea’s ability to (i) reverse the increase in stress-related metabolites (5-HIAA and FFA), (ii) prevent lipid peroxidation (LPO), (iii) restore the stress-induced protein degradation (PD), (iv) regulate glutathione metabolism (GSH and GSH/GSSG ratio), and (v) modulate changes in the activity of antioxidant enzymes (SOD and CAT). However, a series of well-controlled clinical intervention studies are needed to further explore this possibility.
